# Hellbender Genome Sequences Shed Light on Genomic Expansion at the Base of Crown Salamanders

**DOI:** 10.1093/gbe/evu143

**Published:** 2014-06-23

**Authors:** Cheng Sun, Rachel Lockridge Mueller

**Affiliations:** Department of Biology, Colorado State University

**Keywords:** transposable element, genome size evolution, *Cryptobranchus*, indels, age distribution, phylogeny

## Abstract

Among animals, genome sizes range from 20 Mb to 130 Gb, with 380-fold variation across vertebrates. Most of the largest vertebrate genomes are found in salamanders, an amphibian clade of 660 species. Thus, salamanders are an important system for studying causes and consequences of genomic gigantism. Previously, we showed that plethodontid salamander genomes accumulate higher levels of long terminal repeat (LTR) retrotransposons than do other vertebrates, although the evolutionary origins of such sequences remained unexplored. We also showed that some salamanders in the family Plethodontidae have relatively slow rates of DNA loss through small insertions and deletions. Here, we present new data from *Cryptobranchus alleganiensis*, the hellbender. *Cryptobranchus* and Plethodontidae span the basal phylogenetic split within salamanders; thus, analyses incorporating these taxa can shed light on the genome of the ancestral crown salamander lineage, which underwent expansion. We show that high levels of LTR retrotransposons likely characterize all crown salamanders, suggesting that disproportionate expansion of this transposable element (TE) class contributed to genomic expansion. Phylogenetic and age distribution analyses of salamander LTR retrotransposons indicate that salamanders’ high TE levels reflect persistence and diversification of ancestral TEs rather than horizontal transfer events. Finally, we show that relatively slow DNA loss rates through small indels likely characterize all crown salamanders, suggesting that a decreased DNA loss rate contributed to genomic expansion at the clade’s base. Our identification of shared genomic features across phylogenetically distant salamanders is a first step toward identifying the evolutionary processes underlying accumulation and persistence of high levels of repetitive sequence in salamander genomes.

## Introduction

Genome size varies dramatically across species, and the evolutionary forces shaping such variation remain an important research focus across multiple biological disciplines (Gregory 2005; Lynch 2007). Among animals, genome sizes range from approximately 20 Mb to 130 Gb, with 380-fold variation among the vertebrates alone (Gregory 2014). Most of the largest vertebrate genomes are found within the salamanders, a clade of amphibians that includes 660 recognized species (AmphibiaWeb 2014). Salamander genome sizes range from 14 to 120 Gb; these values are larger than all bird, mammal, reptile, and frog genomes, as well as all “fish” genomes with the exception of lungfish (Metcalfe et al. 2012; Metcalfe and Casane 2013; Gregory 2014). Salamanders’ large genomes reflect high levels of repetitive DNA rather than polyploidy (Green 1991; Sessions and Kezer 1991; Batistoni et al. 1995; Marracci et al. 1996), and extensive shared synteny exists between salamanders and other tetrapod vertebrates (Voss et al. 2011).

Because of their unusual genome sizes, salamanders are an important model system for studying the causes and consequences of extreme gigantism in animal genomes (Wake 2009). Historically, most emphasis was placed on examining the phenotypic correlates of genome size; in salamanders, large genomes are correlated with reduced neural complexity and changes to the skeletal and circulatory systems, reflecting increased nucleus and cell sizes and decreased rates of cell division and differentiation (Sessions and Larson 1987; Wiggers and Roth 1991; Hanken and Wake 1993; Roth et al. 1994, 1997; Jockusch 1997; Mueller et al. 2008). More recently, however, advances in sequencing technology have made the identification of sequences that make up these enormous genomes possible (Smith et al. 2005, 2009; Sun, Arriaza, et al. 2012; Sun, Shepard, et al. 2012; Zhu et al. 2012; Voss et al. 2013). Thus, studies targeting the molecular processes underlying genome expansion can now be carried out, complementing earlier approaches focused on organismal phenotype.

In two previous studies, we compared the genomes of six salamander species with five vertebrate genomes of more typical size to generate hypotheses for the molecular processes contributing to genome expansion in salamanders (Sun, Arriaza, et al. 2012; Sun, Shepard, et al. 2012). We showed that these salamander genomes contain all of the main transposable element (TE) superfamilies identified in well-annotated eukaryotic genomes, but that they accumulate much larger amounts of long terminal repeat (LTR) retrotransposons than do other vertebrates (Sun, Shepard, et al. 2012). However, the evolutionary origins of such abundant TEs remained unexplored. We also showed that these salamander genomes have slower rates of DNA loss through small insertions and deletions (indels) than do other vertebrates (Sun, Arriaza, et al. 2012). However, both of these studies were based on sampling from within the salamander family Plethodontidae. Although this family includes over two-thirds of extant salamander diversity (440 of 660 total species of salamanders) (AmphibiaWeb 2014), sampling exclusively within this family precluded us from identifying genomic features shared across all crown salamanders.

Here, we build on these previous studies by generating and analyzing genomic sequence data from *Cryptobranchus alleganiensis* (the hellbender), one of the three species that compose the family Cryptobranchidae. *Cryptobranchus* is a monotypic genus with two morphologically identified subspecies. Cryptobranchidae and Plethodontidae span the basal split within the salamander phylogeny (Pyron and Wiens 2011); thus, by integrating analyses of *Cryptobranchus* and members of the family Plethodontidae, we can make inferences about the genome of the ancestral crown salamander lineage, which underwent genome expansion. Our results indicate that high levels of LTR retrotransposons likely characterize the entire crown salamander clade, suggesting that disproportionate expansion of this class of TEs is one contributor to genomic expansion. In addition, both phylogenetic and age distribution analyses of the most abundant LTR retrotransposon superfamily (Gypsy/Ty3) suggest that high TE levels in salamanders reflect persistence and diversification of ancestral TE families within salamander lineages rather than horizontal transfer from distant branches of the Tree of Life. Finally, our results indicate that relatively slow rates of DNA loss through small indels likely characterize the entire crown salamander clade, suggesting that a decreased DNA loss rate is another contributor to genomic expansion at the base of crown salamanders.

## Materials and Methods

### Specimen Information

We sequenced a DNA sample from a single captive adult male *C. alleganiensis* from the Eleven Point River in northern Arkansas, obtained from the St. Louis Zoo (C95DM). *Cryptobranchus alleganiensis* has a genome size of approximately 55 Gb (Tiersch and Chandler 1989; Gregory 2014).

### Shotgun Library Creation and Sequencing

Total DNA was extracted from heart, liver, and kidney tissues stored in RNA Later using the PureGENE DNA extraction kit (Qiagen). 454 FLX–LR shotgun libraries were prepared using the 454 shotgun library preparation kits and protocols (Roche) for FLX sequencing. Libraries were sequenced on the Roche 454-FLX platform with XLR 70 Titanium reagents, allocating one sequencing plate. Library preparation and sequencing were performed by the University of Idaho Institute for Bioinformatics and Evolutionary Studies (IBEST) Genomics Resources Core facility.

### Initial Data Processing

Shotgun reads were checked for sequencing artifacts generated by the presence of multiple beads and a single template in emPCR drops, which can potentially produce multiple identical sequences that can skew estimates of repeat element abundance (Gomez-Alvarez et al. 2009; Niu et al. 2010). The locally installed cdhit-454 (Li and Godzik 2006) was used to filter out replicates, with default parameters.

### Mining and Classification of Repeat Elements

We modified a pipeline used in our previous studies (Sun, Shepard, et al. 2012) to mine and classify repeats from low-coverage genomic shotgun data in taxa that lack genomic resources. The pipeline included the following steps: 1) RepeatScout (Price et al. 2005) was used to identify de novo repeats from shotgun reads, with default parameters. Identified repeats that were ≤50 nt or >50% low-complexity were filtered out. 2) Shotgun reads were assembled into contigs using Newbler (http://contig.wordpress.com/table-of-contents/, last accessed July 3, 2014) with default parameters. To identify contigs that represent TEs, contig sequences were used as queries to BLASTx against the amino acid sequences of TE-encoded proteins (http://www.repeatmasker.org/RepeatProteinMask.html#database, last accessed July 3, 2014), with an *e* value threshold cutoff of 1e-10. Contigs representing TEs were refined manually to avoid assembly artifacts (Sun, Shepard, et al. 2012). 3) Repeats identified in step (1) were classified using BLASTn against the TE contigs identified in step (2), with an *e* value cutoff of 1e-5. Remaining unclassified repeats were used as queries to tBLASTx against the most recent release of RepBase (RepBase16.12), with an *e* value threshold of 1e-5. 4) All classified repeats, along with the unclassified repeats (referred to as “unknown repeats” hereafter), were combined to produce a hellbender-specific repeat library. Using this library, we masked the shotgun reads with RepeatMasker (http://www.repeatmasker.org/, last accessed July 3, 2014). Simple repeats were identified using the Tandem Repeat Finder module (http://tandem.bu.edu/trf/trf.html, last accessed July 3, 2014), implemented in RepeatMasker. Repeats were classified as 1) TEs belonging to known superfamilies (“known TEs,” collectively), 2) unknown repeats, and 3) simple repeats. In an effort to identify more highly divergent repeat element copies, we used the element-specific P-cloud approach (Gu et al. 2008; de Koning et al. 2011), with default parameters, to build two sets of oligo clouds: one from all the known TEs we identified, and a second from all the unknown repeats we identified. These two sets of oligo clouds were then used to reannotate the shotgun reads that our initial pipeline recognized as nonrepetitive, searching for additional repeat copies. Additionally, the set of oligo clouds built from the known TEs was used to reannotate the shotgun reads that our initial pipeline identified as unknown repeats, searching for additional copies of known TEs.

### Comparison of *Cryptobranchus* TE Levels with Other Vertebrate Genomes

Our previous work identified high levels of LTR retrotransposons in plethodontid salamander genomes relative to the genomes of other vertebrates with more typical genome sizes (Sun, Shepard, et al. 2012). To test whether the high levels of LTR retrotransposons in plethodontid salamander genomes are likely to be characteristic of all crown group salamander genomes, we calculated the relative frequency and the total Gb of the *C. alleganiensis* genome composed of LTR retrotransposons and compared them with those estimated from six species of plethodontid salamanders (*Aneides flavipunctatus*, *Batrachoseps nigriventris*, *Bolitoglossa occidentalis*, *Bo. rostrata, Desmognathus ochrophaeus*, and *Eurycea tynerensis*; genome sizes range from ∼15 to ∼44 Gb) as well as five vertebrates with more typical genome sizes (*Homo sapiens*, *Gallus gallus*, *Danio rerio*, *Anolis carolinensis*, and *Xenopus tropicalis*; genome sizes range from ∼1.25 to ∼3 Gb). To make the results comparable across all seven species of salamanders, we reanalyzed the plethodontid salamander genomic shotgun data sets using the modified pipeline presented here. We also compared estimates of individual TE frequencies among *Cryptobranchus* and the six species of plethodontid salamanders to examine how repeat landscape composition varies across crown salamanders. To verify that approximately 1% shotgun coverage gives sufficiently accurate estimates of overall TE content to support the main conclusions of this study, we analyzed five human genome 454 sequencing runs (accession numbers: SRR000445, SRR000522, SRR000549, SRR000554, and SRR000564), each of which has a sequencing coverage of approximately 1%, using the same pipeline used for our salamander shotgun data and compared the results with those obtained using the complete human genome. Of the five nonsalamander vertebrates that we examined in this study, the human genome has an LTR retrotransposon level most similar to our salamander estimates (Sun, Arriaza, et al. 2012), although the genome is much smaller, increasing the effects of stochastic sampling error relative to 1% coverage of salamander genomes.

### Evolutionary Origins of the Most Abundant Salamander LTR Retrotransposons

To determine whether the most abundant salamander TEs increased in frequency because of 1) multiple horizontal transfer events into salamander genomes, or 2) persistence and/or proliferation of TE lineage(s) within salamander genomes, we estimated the phylogenetic tree for sequences of the most abundant salamander TE superfamily (Gypsy/Ty3) from salamanders as well as other organisms. Because we are working with low-coverage genomic shotgun data, we limited our analyses to the amino acid sequences of the highly conserved Ribonuclease H (RNase H) domain to avoid problems introduced by the presence of missing data and alignment ambiguity (Malik and Eickbush 2001). First, the Gypsy/Ty3-derived consensus sequences of each salamander species were used as queries to BLASTx against a protein database comprised the protein sequences used to construct the RNase H domains of Ty3/Gypsy reverse transcriptase in the NCBI (National Center for Biotechnology Information) Conserved Domain Database (accession number cd09274), with an *e* value cutoff of 1e-10 and a hit length cutoff of 100 amino acids. Second, the translated salamander consensus sequences with significant hits to this database were trimmed to the RNase H domain and then clustered by CD-HIT (http://weizhong-lab.ucsd.edu/cdhit_suite/cgi-bin/index.cgi?cmd=cd-hit, last accessed July 3, 2014), with a sequence identity cutoff of 80%, to reduce redundancy in the data set; representative sequences were kept for future phylogenetic analysis. Third, to identify related RNase H domains in other species, these salamander RNase H domains were used as queries to BLASTp against the transposition-related protein database (http://www.repeatmasker.org/RepeatProteinMask.html#database, last accessed July 3, 2014), with an *e* value cutoff of 1e-5. The top three hits for each query were combined with the salamander sequences for phylogenetic analysis. Amino acid sequences were aligned using MUSCLE (Edgar 2004) implemented in MEGA 5.0 (Tamura et al. 2011). Alignments were checked manually to eliminate ambiguously aligned regions. The final alignment included 92 OTUs and 116 amino acid positions. We estimated the phylogeny using maximum likelihood, implemented in MEGA 5.0, using the best-fitting model of amino acid substitution (rtREV + G). Gaps were partially deleted, with a threshold of 90%. Bootstrap support for nodes was calculated using 500 bootstrap replicates. We estimated an unrooted tree because the large evolutionary distances separating our focal clade of RNase H domain sequences from any relevant outgroup (e.g., the RNase H domain of Ty1) made accurate alignment impossible. The alignments and resulting trees are deposited in TreeBASE (www.treebase.org, last accessed July 3, 2014) (TB2 ID number: 14376).

### Proliferation Dynamics of the Most Abundant Salamander LTR Retrotransposons

To summarize historical patterns of proliferation of the most abundant TE superfamily (Gypsy/Ty3) in the crown salamander clade, we calculated the age distributions of such elements in *Cryptobranchus* and four plethodontid salamanders for which we have approximately 1% sequencing coverage (Sun, Shepard, et al. 2012). First, for each species, we used RepeatScout to estimate a consensus sequence from all genomic copies of each Gypsy/Ty3 family/subfamily; this consensus represents the master gene (i.e., ancestral) repeat sequence for the family/subfamily. Second, we used the consensus sequences from each species to mask the corresponding genomic shotgun data set with RepeatMasker (http://www.repeatmasker.org/, last accessed July 3, 2014), generating pairwise alignment files of ancestral and descendant repeat element copies. From these pairwise alignments, we estimated sequence divergence, correcting with the Jukes–Cantor model of nucleotide substitution. However, some of the sequences that we identified as confamilial were likely generated by multiple active master genes that differed from one another in sequence. In such a case, a single consensus sequence would not accurately represent the ancestral state of all individual element copies; some of the differences between ancestor and descendant sequences would correspond to substitutions that occurred along the active master element lineages. This would produce upwardly biased estimates of sequence divergence from consensus. To minimize this problem, we parsed the RepeatMasker-generated pairwise alignments to identify groups of two or more substitutions shared by two or more Gypsy/Ty3 copies, as such shared substitutions likely accumulated in the active lineages (Price et al. 2004). We excluded these substitutions from our estimates of sequence divergence between ancestral and descendant repeat element copies, producing refined estimates of sequence divergence. This process was automated using in-house Perl scripts, which are available upon request. To increase accuracy, we limited our analysis to Gypsy/Ty3 copies greater than 100 bp in length and ≥80% identical to their estimated consensus. Finally, we plotted the percentage of total shotgun reads as a function of sequence divergence from consensus; assuming equal rates of nucleotide substitution, such sequence divergence distributions are a proxy for age distributions.

### Comparison of *Cryptobranchus* DNA Loss Rates with Other Vertebrate Genomes

Our previous work identified slower rates of DNA loss through small (≤30 bp) indels in plethodontid salamanders than in other vertebrates (Sun, Arriaza, et al. 2012). To test whether slower DNA loss rates are likely to be a common feature of all crown group salamander genomes, we estimated the DNA loss rate in *Cryptobranchus* using our previously published methods for low-coverage genomic shotgun data sets (Sun, Arriaza, et al. 2012). Briefly, we generated consensus sequences for all non-LTR retrotransposon families using RepeatScout and trimmed such sequences to the protein-coding regions. We then used these consensus sequences to mask the shotgun reads with RepeatMasker to generate pairwise alignment files. Based on the obtained alignments, we eliminated all non-LTR sequences with nonrandom distributions of substitutions across codon positions (χ2 test; *P* < 0.05) to avoid counting substitutions that occurred along master element lineages (Petrov et al. 1996). For each remaining non-LTR element copy (31,920 copies in total), the numbers of insertions, deletions, and substitutions (after Jukes–Cantor correction) relative to the ancestral sequence were obtained based on the RepeatMasker-generated alignment, and the sums of these values for every individual element copy were used to represent the total amounts of DNA gained and lost through small indels in *Cryptobranchus* (bp deleted − bp inserted/substitution). Based on these alignments, we also calculated the numbers of individual insertion and deletion events, as well as the size of each insertion and deletion. 454 sequencing is error-prone in homopolymer regions, producing length ambiguity that could be interpreted as indels; we minimized this problem by focusing our analyses on ORFs, and we verified that the indels we counted were not within long homopolymer stretches. We compared DNA loss rates in *Cryptobranchus* with loss rates in four plethodontid salamanders for which we have approximately 1% sequencing coverage (*Ane. flavipunctatus*, *Ba. nigriventris*, *De. ochrophaeus*, and *E. tynerensis*) as well as five vertebrates with more typical genome sizes (*H. sapiens, G. gallus, Da. rerio, Ano. carolinensis*, and *X. tropicalis*).

To verify that approximately 1% shotgun coverage gives sufficiently accurate estimates of DNA loss rates to support the main conclusions of this study, we also analyzed five approximately 1% coverage human genome 454 sequencing runs (those used in our analyses of TE content). Of the five nonsalamander vertebrates that we examined in this study, the human genome has a DNA loss rate most similar to our salamander estimates (Sun, Arriaza, et al. 2012), although the smaller genome increases the effects of sampling error. We performed these analyses using the same methods used for our salamander shotgun data. In addition, we calculated DNA loss rates using methods that masked the human 454 shotgun reads with all curated human non-LTR retrotransposon consensus sequences from RepBase rather than just the consensus sequences generated by our pipeline using RepeatScout; we did this to produce DNA loss rate estimates that encompassed the diversity of non-LTR retrotransposon families present in the human genome (see Results). Finally, we compared the results of these analyses with those obtained using the complete human genome.

## Results

### Shotgun Library Summary and Initial Data Processing

In total, 2,057,025 shotgun reads were obtained from *Cryptobranchus,* with a total length of 838,596,655 bp. Sequences have been deposited in the GenBank sequence read archive (accession number: SRA073787). After filtering out potential sequencing artifacts, 1,728,346 shotgun reads remained, with a total length of 732,630,628 bp. Based on the estimated 55 Gb genome size for *Cryptobranchus* (Tiersch and Chandler 1989; Gregory 2014), the sequencing coverage is approximately 1.33%.

### Repeat Content of the *Cryptobranchus* Genome

**Fig. 1.—:**
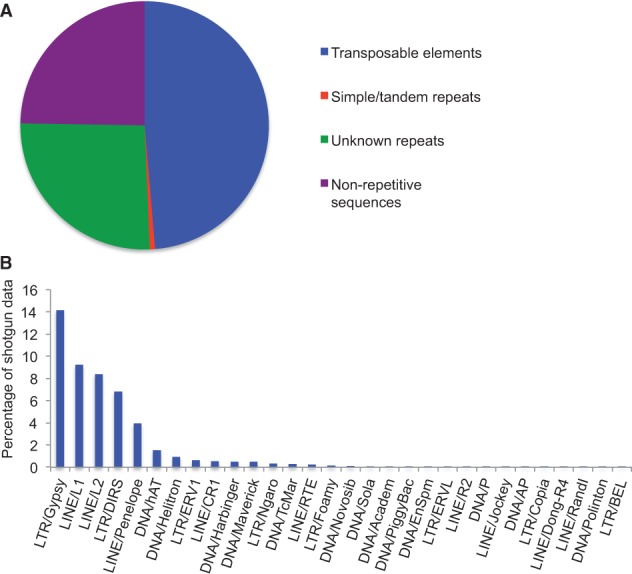
(*A*) Pie-chart summarizing the proportions of the *Cryptobranchus* genome identified by our repeat-mining pipeline as TEs, simple/tandem repeats, unknown repeats, and nonrepetitive sequences. The majority of the genome is repetitive. (*B*) Percentage of the shotgun data (bp) identified as TEs from different superfamilies.

Over 75% of the *Cryptobranchus* shotgun data (in bp) was recognized as repetitive sequences (fig. 1*A*) by our repeat-mining pipeline. The majority of such repeats (∼49% of the shotgun data) are known TEs; in contrast, simple/tandem repeats compose only a tiny fraction (0.69%), and unknown (nonsimple, nontandem within the length of a single read) repeats compose approximately 26% (fig. 1*A*). We identified 30 TE superfamilies (fig. 1*B* and supplementary file S1, Supplementary Material online), demonstrating that the *Cryptobranchus* genome includes most of the major TE types reported in previously characterized eukaryotic genomes. The identified TE superfamilies are ranked by abundance in figure 1*B*. LTR/Gypsy is the most abundant superfamily (14.1% of the data set), followed by LINE/L1 (9.2%), LINE/L2 (8.4%), LTR/DIRS (6.8%), and LINE/Penelope (3.9%); together, these five most abundant TE superfamilies account for 42.4% of the genome, assuming that shotgun data are a random representation of the whole genome. P-cloud analysis identified an additional 0.05% of the nonrepetitive sequences (0.01% of the total data set) as repetitive and 5% of the unknown repeats (1.3% of the data set) as known TEs. Because these additional annotations had little impact on our overall repeat classification, we focus our analysis and interpretation on the results from our repeat-mining pipeline.

### Comparison of *Cryptobranchus* TE Levels with Other Vertebrate Genomes

**Fig. 2.—:**
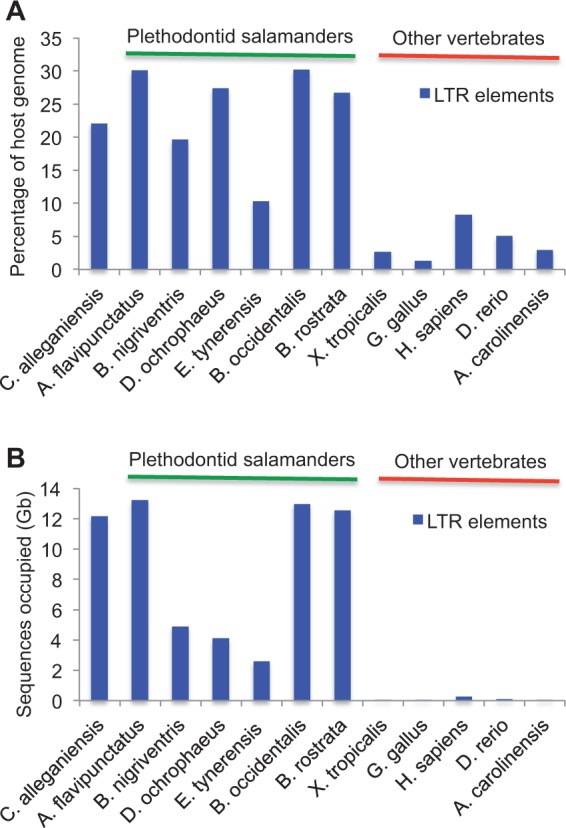
(*A*) Levels of LTR retrotransposons in the *Cryptobranchus* genome, as well as the genomes of six plethodontid salamanders and five vertebrates with typical genome sizes, shown as the percentage of the host genome (bp). (*B*) Levels of LTR retrotransposons in the *Cryptobranchus* genome, as well as the genomes of six plethodontid salamanders and five other vertebrates, shown as Gb. *Cryptobranchus*, as well as the plethodontid salamanders, has high levels of LTR retrotransposons relative to other vertebrates. Analyses of human genome 454 sequence data sets comparable to our salamander sequencing coverage (i.e., ∼1%) suggest that 1% shotgun data produce underestimates of LTR levels; thus, the differences we report here are likely conservative.

The percentages of the genome composed of LTR retrotransposons in *Cryptobranchus*, six species of plethodontid salamanders, and five vertebrates with more typically sized genomes are shown in figure 2. The *Cryptobranchus* genome has LTR retrotransposon levels similar to those found in other salamanders, which are much higher than the levels found in nonsalamander vertebrates. LTR retrotransposon levels estimated from the five human 1% coverage 454 runs range from 2.36% to 3.03%, all of which are lower than the value (8.3%) estimated from the complete human genome (supplementary file S2, Supplementary Material online). This is consistent with our expectations, as the analysis of low-coverage shotgun data underestimates true TE content in predictable ways: 1) We miss low-copy-number TE families, and 2) we miss some sequences, especially the noncoding portions, from older/divergent TE families (Metcalfe et al. 2012; Sun, Shepard, et al. 2012). Thus, our salamander estimates are likely underestimates of the true LTR retrotransposon content, suggesting that our reported difference in genome content between salamanders and other taxa is conservative. Based on these results, we suggest that high levels of LTR retrotransposons were likely present in the ancestral crown salamander lineage and retained in the lineages sampled in our study, contributing to genomic expansion. The most abundant retrotransposon superfamily in *Cryptobranchus*—Gypsy/Ty3—is the most abundant superfamily in all seven species of salamanders we have examined (fig. 1*B* and supplementary file S1, Supplementary Material online). The second and third most abundant LTR superfamilies in *Cryptobranchus*—DIRS and ERV1—are also in the top three most abundant LTR elements in all examined salamander genomes (supplementary file S1, Supplementary Material online).

**Fig. 3.—:**
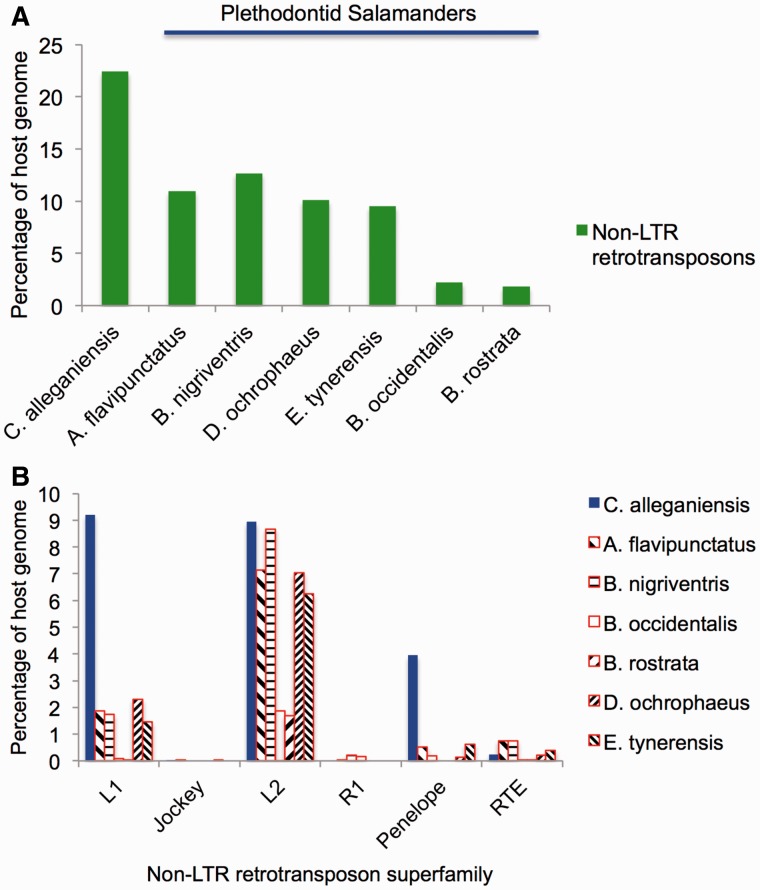
(*A*) Levels of non-LTR retrotransposons in the *Cryptobranchus* genome, as well as the genomes of six plethodontid salamanders. *Cryptobranchus* has higher levels of non-LTR retrotransposons than do the other salamander species. (*B*) Levels of six abundant non-LTR retrotransposon superfamilies in the *Cryptobranchus* genome, as well as the genomes of six plethodontid salamanders. L1 and Penelope are much more abundant in the *Cryptobranchus* genome.

The *Cryptobranchus* genome and the six plethodontid salamander genomes have similar/overlapping levels of DNA transposons (supplementary file S3, Supplementary Material online), and most of the seven salamander genomes share the same four most abundant DNA transposons—DNA/hAT, DNA/Helitron, DNA/Harbinger, and DNA/Maverick (supplementary file S1, Supplementary Material online). In contrast, non-LTR retrotransposons are more than twice as abundant in the *Cryptobranchus* genome than in plethodontid salamander genomes (fig. 3*A*). More specifically, LINE/L1 and LINE/Penelope elements are much more abundant in *Cryptobranchus* than they are in plethodontid salamanders (fig. 3*B*). LINE/L2 elements, the most abundant non-LTR retrotransposon in plethodontid salamanders, are present at comparable levels in *Cryptobranchus.*

### Evolutionary Origins of the Most Abundant Salamander LTR Retrotransposons

BLASTx of the salamander Gypsy/Ty3-derived consensus sequences against an RNase H domain database identified 115 salamander consensus sequences (8–47 sequences per salamander species) that include the RNase H domain. Following clustering with CD-HIT to reduce redundancy in the data set, 51 RNase H sequences from salamanders were initially retained; in all cases, sequences clustered with others from the same species, indicating that elements have diversified within each salamander lineage. BLASTp of these RNase H sequences against the transposition-related protein database retrieved 47 additional sequences from 14 different taxa (22 total sequences from five vertebrate taxa, 20 total sequences from six invertebrate taxa, and three sequences, one from each of three plant taxa). Six sequences (four from salamanders, two from other taxa) were eliminated from further analysis because of their short length.

**Fig. 4.—:**
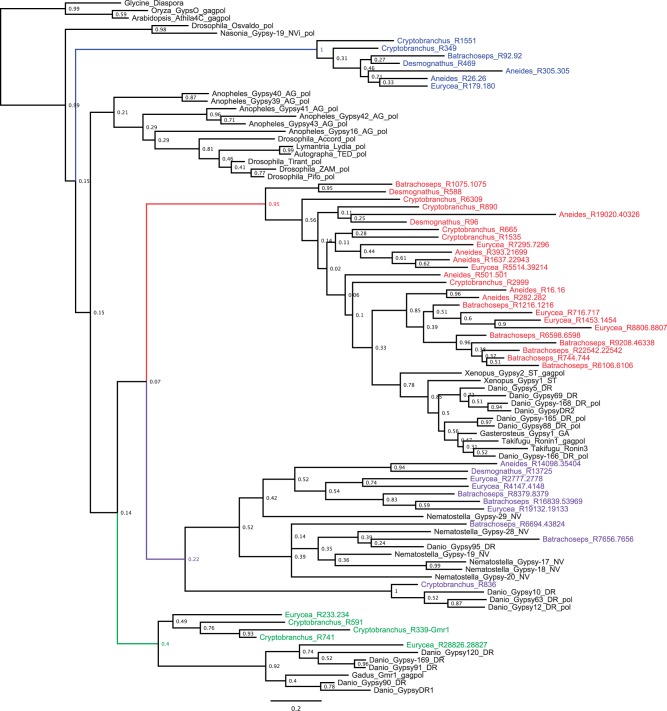
Maximum likelihood tree estimated from aligned amino acid sequences of the RNase H domain from Gypsy/Ty3 retrotransposons in salamanders and other taxa. Tip names include genus name followed by retrotransposon family/subfamily name. Bootstraps above 50% are shown. Salamander sequences, and the ancestral lineages of the clades that contain salamander sequences, are indicated by color. The tree is unrooted. The majority of the salamander sequences are most closely related to other vertebrate sequences.

The maximum-likelihood phylogeny estimated from this data set is presented in figure 4. The Gypsy/Ty3 elements found in salamander genomes fall within four clades, indicated by different colors, three of which are paraphyletic with respect to Gypsy/Ty3 elements found in other taxa. The largest group of salamander Gypsy/Ty3 elements (base of the clade and salamander lineages highlighted in red), composed of 25 of 47 salamander elements, is paraphyletic with respect to ten fish (*Danio*, *Takifugu*, *Gasterosteus*) and two frog (*Xenopus*) elements. The second largest group (highlighted in purple) includes 10 of 47 salamander Gypsy/Ty3 elements, three *Danio* elements, and six elements from *Nematostella vectensis* (sea anemone). The third largest group (highlighted in green) includes five salamander elements and six fish (*Danio, Gadus*) elements. The final clade of salamander RNase H domain sequences (seven elements, highlighted in blue) shows high sequence divergence from all remaining elements. Many nodes, particularly those deeper in the tree, show low bootstrap support values, reflecting the low phylogenetic signal in the alignment (i.e., relatively few amino acid positions, ancient evolutionary divergences, and a highly conserved domain). Thus, we interpret these results with caution. Overall, our results are not consistent with rampant horizontal transfer into the salamander clade from distant species; rather, they are consistent with persistence and/or proliferation of TE lineage(s) within salamander genomes.

### Proliferation Dynamics of the Most Abundant Salamander LTR Retrotransposons

**Fig. 5.—:**
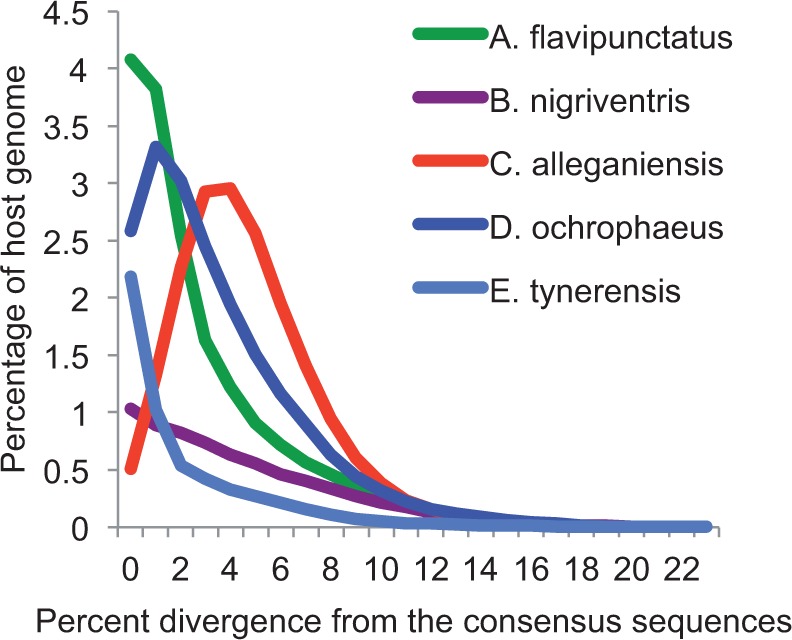
Sequence divergence distributions for Gypsy/Ty3 elements in the *Cryptobranchus alleganiensis* genome, as well as four species of plethodontid salamanders. Distributions for *Aneides flavipunctatus*, *Eurycea tynerensis*, and *Batrachoseps nigriventris* suggest ongoing proliferation. Distributions for *Desmognathus ochrophaeus* and *C. alleganiensis* suggest a peak of proliferation in the past and decreased activity toward the present. The presence of a single peak in all species is inconsistent with multiple horizontal transfer events into salamander genomes.

The sequence divergence (i.e., age) distributions for Gypsy/Ty3 elements for five species of salamanders (*Cryptobranchus* and four plethodontid species) are shown in figure 5. In three species (*Ane. flavipunctatus*, *E. tynerensis,* and *Ba. nigriventris*), the distribution suggests extensive ongoing proliferation, indicated by high relative frequencies of element copies with sequence divergence ≤1% from the consensus (Novick et al. 2010). In the remaining two cases (*Cryptobranchus* and *De. ochrophaeus*), sequences ≤1% divergent from the consensus exist, but the shape of the distribution suggests a peak of proliferation in the past with reduced activity toward the present. This is particularly apparent for *Cryptobranchus* (fig. 5)*.* Thus, although high levels of Gypsy/Ty3 elements are present in both Cryptobranchidae and Plethodontidae, suggesting that they are likely shared across crown salamanders, the proliferation dynamics of this TE superfamily differ among species. Despite these differences, however, only a single peak exists in all five species. This pattern is inconsistent with multiple horizontal transfer events into salamander genomes during the relatively recent past, corroborating the results from our phylogenetic analysis.

### Comparison of *Cryptobranchus* DNA Loss Rates with Other Vertebrate Genomes

**Fig. 6.—:**
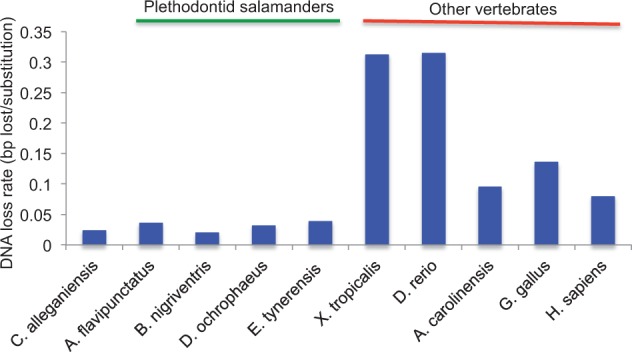
DNA loss rate (bp deleted − bp inserted/substitutions) from *Cryptobranchus*, four species of plethodontid salamanders, and five nonsalamander vertebrates with typical genome sizes. *Cryptobranchus*, as well as the plethodontid salamanders, has lower rates of DNA loss than the nonsalamander vertebrates. Analyses of human genome 454 sequence data sets comparable to our salamander sequencing coverage (i.e., ∼1%) suggest that 1% shotgun data produce less accurate estimates of DNA loss rate than whole-genome analyses; thus, the extent of the difference between salamanders and other vertebrates that we report should be interpreted with caution.

The DNA loss rates from the *Cryptobranchus* genome, as well as from four species of plethodontid salamanders and five nonsalamander vertebrate species, are shown in figure 6. In total, we detected 39,258 insertions, 29,755 deletions, and 658,447 substitutions from 31,920 copies of non-LTR retrotransposon sequences in the *Cryptobranchus* genome. The DNA loss rate in *Cryptobranchus* is 0.024 bp/substitution, which is comparable to that estimated previously from four species of plethodontid salamanders. This rate is lower than the rates estimated in our five focal nonsalamander vertebrates (fig. 6). The mean deletion and insertion sizes in the *Cryptobranchus* genome are 2.60 and 1.47 bp, respectively; these are comparable to the deletion sizes in plethodontids and smaller than the sizes in nonsalamander vertebrates (Sun, Arriaza, et al. 2012). The ratio of the number of deletions to the number of insertions in the *Cryptobranchus* genome is 0.76; this ratio, like that in plethodontid salamanders, is lower than that in nonsalamander vertebrates (Sun, Arriaza, et al. 2012).

When we estimated DNA loss rates from the five human 1% coverage 454 runs using methods identical to those we used for salamanders, we obtained consensus sequences for no non-LTR retrotransposon families for two such data sets (making DNA loss rate estimates unobtainable) and consensus sequences for only one or two families for the remaining three such data sets. One or two families is a much lower number than we obtained from our salamander shotgun data sets (Sun, Arriaza, et al. 2012), suggesting that the empirically determined criteria we applied to generate accurate salamander consensus sequences (i.e., >330 bp in overall length and estimated from at least five element copies, each of which was >300 bp in length and ≥80% identical to the estimated consensus) were too conservative for the human genome based on 1) the diversity and divergence of human non-LTR retrotransposon families, and 2) the much smaller size of the human genome (and, by extension, the smaller size of the 1% subsampled data sets). More generally, one or two families is a very small fraction of the total human non-LTR retrotransposon landscape (http://www.girinst.org/, last accessed July 3, 2014) (Sun, Arriaza, et al. 2012). Because DNA loss rate varies by family, analyzing such a low number of families would fail to produce a DNA loss rate estimate that encompasses genome-wide diversity, making the results incomparable with our salamander shotgun results. Thus, we used RepBase-curated human non-LTR retrotransposon consensus sequences in the early read-masking stage of our pipeline, producing DNA loss rate estimates from the five human data sets for 163 non-LTR retrotransposon families. Such estimates differ, on average, by 17% from the estimates obtained using the complete human genome data set (range = 2–34%, including four underestimates and one overestimate produced by shotgun data) (supplementary file S2, Supplementary Material online). For all five human 1% coverage data sets, DNA loss rates are higher than the DNA loss rates estimated from 1% salamander shotgun data sets (supplementary file S4, Supplementary Material online). Taken together, these results suggest that, although 1% shotgun data sets 1) produce less accurate estimates than whole genomes, and 2) may be more likely to yield underestimates than overestimates, the use of shotgun data is not likely to produce an artifact of greater than 2-fold lower DNA loss rates in five species of salamanders than in five other vertebrates. Thus, we conclude that *Cryptobranchus* and plethodontids have lower rates of DNA loss through small indels than other vertebrates, although the extent of the differences between salamanders and other vertebrates that we report should be interpreted with caution. These results suggest that a decreased rate of DNA loss through small indels, reflecting fewer and smaller deletion events, was present in the ancestral crown salamander lineage and retained in the lineages sampled in our study, contributing to genomic expansion. Our methods do not measure large indels, which also contribute to evolutionary changes in genome size.

## Discussion

Our results identify several genomic features shared across phylogenetically distant salamander species. These shared features, in turn, likely characterized the genome of the ancestral crown salamander lineage, which underwent genomic expansion; however, we cannot eliminate the less parsimonious alternative that these shared features evolved independently in Cryptobranchidae and Plethodontidae without sampling additional salamander lineages. We show that high levels of LTR retrotransposons were likely present in the ancestral crown salamander lineage, and that these LTR retrotransposons are vertically transmitted, persisting and proliferating within crown salamanders. We also show that a decreased rate of DNA loss through small indels, reflecting fewer and smaller deletions, was likely present in the ancestral crown salamander lineage. In contrast to these shared characteristics, we also identify differences in genome composition across crown salamanders, suggesting variation in the balance between TE proliferation and silencing among species. Our identification of shared genomic features across phylogenetically distant salamanders, despite differences in genome size and composition, is a first step toward identifying the evolutionary processes that contributed to the accumulation and persistence of unusually high levels of repetitive sequence in salamander genomes.

### TE Proliferation and Genomic Expansion

The majority of salamander Gypsy/Ty3 elements are found within clades that contain other salamander sequences as well as sequences from other vertebrate genomes (i.e., fish, frog) (fig. 4). The basal relationships within such clades are poorly supported; thus, we typically cannot infer with confidence whether salamander elements are paraphyletic with respect to these other taxa or whether salamander elements, frog elements, and fish elements are all reciprocally monophyletic. These two topologies imply different evolutionary origins for the TE lineages within salamanders; for example, salamander paraphyly with respect to fish sequences would suggest that elements that predate the split between ray-finned fishes and tetrapods have remained active in salamanders, but have been lost from fish genomes. Alternatively, reciprocal monophyly of sequences from fishes, frogs, and salamanders (i.e., a TE tree that matches the species tree) would suggest that the TEs found in salamanders began to diversify within the ancestral salamander lineage after it diverged from the ancestral frog lineage. Given the weak support for these relationships, we conservatively infer that ancestral vertebrate TE lineages have remained active in salamanders, despite being inactivated in other vertebrate genomes, and/or have diversified within the salamander clade.

One group of salamander Gypsy/Ty3 elements is found within the same clade as sequences from both *Da. rerio* (zebrafish, another vertebrate) and *N. vectensis* (sea anemone). Although there is an ongoing debate about the relationships at the base of the metazoan tree, cnidarians (including sea anemones) and bilaterians (including vertebrates) last shared a common ancestor many hundreds of millions of years ago (Nosenko et al. 2013); thus, this result most likely reflects convergence in amino acid sequence between salamanders and *N. vectensis.* Other possible explanations are less plausible; horizontal transfer between salamanders and sea anemones, which occupy different ecosystems (terrestrial and marine), is unlikely, as is widespread loss of this TE lineage from all other metazoan taxa.

### DNA Loss and Genomic Expansion

DNA loss rates in salamanders are slower than in the nonsalamander vertebrates we examined; however, because substantial variation in rates exists among these nonsalamander species, the difference in DNA loss rate between salamanders and other vertebrates varies depending on taxa compared. In *Cryptobranchus*, as in plethodontid salamanders, slower rates of DNA loss reflect fewer and smaller deletion events per substitution than are found in other vertebrate taxa (Sun, Arriaza, et al. 2012). Indels ≤ 30 bp in length have long been attributed to uncharacterized errors in DNA replication and/or recombination (Petrov et al. 1996; Kvikstad et al. 2007). Recently, comparative genomic analyses have begun to leverage natural variation in indel dynamics, across both genomes and lineages, to reveal the specific mechanisms of indel formation (Kvikstad et al. 2007, 2009; Hu et al. 2011; Nam and Ellegren 2012). Such analyses suggest that the molecular mechanisms producing indels are partially distinct from one another, despite some overlap (Ball et al. 2005; Kvikstad et al. 2007, 2009; Messer and Arndt 2007; Tanay and Siggia 2008). Because salamanders experience fewer deletions than do other vertebrates, deletion-generating processes are candidates to explain salamanders’ slower rates of DNA loss (Sun, Arriaza, et al. 2012). These processes include 1) recombination associated with meiotic crossing over, and 2) the introduction and repair of double-strand DNA breaks (Kvikstad et al. 2009; Nam and Ellegren 2012). Additionally, because both deletion and insertion sizes are smaller in salamanders than in other vertebrates, processes generating both kinds of indels are also candidates to explain salamanders’ slower rates of DNA loss. These include DNA replication and the repair of paused replication forks (Kvikstad et al. 2009). Because accurate estimates of absolute neutral substitution rates are lacking in salamanders, quantifying this contribution to genomic expansion (Gregory 2004) is not feasible with confidence. However, our focal salamanders encompass a 3.6-fold difference in genome size not explained by variation in DNA loss rate, and *G. gallus*, which has the smallest genome, has an intermediate rate of DNA loss among the nonsalamander vertebrates (fig. 6). These patterns suggest that DNA loss rate through small indels contributes to genome size differences among vertebrates (e.g., salamanders vs. other lineages), but that it is not the sole determinant of genome size (Gregory 2004; Sun, Arriaza, et al. 2012).

Despite likely shared genomic features across crown salamanders (i.e., high levels of LTR retrotransposons, lower rates of DNA loss through small indels), differences in genome composition do exist between *Cryptobranchus* and the plethodontid salamanders. Specifically, L1 and Penelope non-LTR retrotransposons are far more abundant in the *Cryptobranchus* genome than in plethodontids (fig. 3). Differences also exist in the amounts of LTR retrotransposons and DNA transposons, as well as overall genome size, across crown salamanders (fig. 2, supplementary file S2, Supplementary Material online). Finally, differences exist in the proliferation histories of Gypsy/Ty3 elements, the most abundant TE superfamily, across crown salamanders (fig. 5). Thus, our results indicate that the balance between proliferation of different TE classes and host silencing and/or removal differs not only between salamanders and other vertebrates, but across crown salamanders as well. If reduced TE suppression/removal evolved at the base of crown salamanders, as our data suggest, then all salamander lineages inherited this reduced suppression/removal from a common ancestor. Over the subsequent ≥ 200 Myr of salamander evolution (Mueller 2006; Marjanović and Laurin 2007; Wiens 2007; Pyron 2011), genome content within individual salamander lineages has evolved along its own evolutionary trajectory, reflecting unique interactions among mutation (e.g., new TE insertions), selection (e.g., on TE silencing machinery, or on individual TE insertions), and genetic drift.

## Conclusions

Our study used newly generated genomic shotgun sequence data from the basal salamander lineage *C. alleganiensis*, in combination with previously published data from plethodontid salamanders, to identify genomic features likely to be shared across all crown salamanders. Despite differences in genome size and content across such species, we show that 1) disproportionately high levels of LTR retrotransposons, which have persisted and diversified within salamander lineages, and 2) a slower rate of DNA loss, resulting from smaller numbers of deletions and smaller deletion size, are likely shared across crown salamanders. Our identification of such shared genomic features across phylogenetically distant salamanders is a first step toward identifying the evolutionary processes that contributed to the accumulation and persistence of unusually high levels of repetitive sequence in salamander genomes.

## Supplementary Material

Supplementary files S1–S4 are available at *Genome Biology and Evolution* online (http://www.gbe.oxfordjournals.org/).

Supplementary Data
