# Supplementary material for: Hellbender Genome Sequences Shed Light on Genomic Expansion at the Base of Crown Salamanders
Source: Genome Biol Evol. 2014 Jun 23;6(7):1818–29. doi: 10.1093/gbe/evu143 (PMC4122941; doi:10.1093/gbe/evu143)
Supplement: Supplementary Data [file supp_6_7_1818__index.html]

Hellbender genome sequences shed light on genomic expansion at the base of crown salamanders — Hellbender Genome Sequences Shed Light on Genomic Expansion at the Base of Crown Salamanders — Supplementary Data 

# Hellbender Genome Sequences Shed Light on Genomic Expansion at the Base of Crown Salamanders

## Supplementary Data

files

**Files in this Data Supplement:**

- Supplementary Data - pdf file
- Supplementary Data - pdf file
- Supplementary Data - xlsx file
- Supplementary Data - xlsx file
