# Supplementary figures and images for: Hellbender Genome Sequences Shed Light on Genomic Expansion at the Base of Crown Salamanders
Source: Genome Biol Evol. 2014 Jun 23;6(7):1818–29. doi: 10.1093/gbe/evu143 (PMC4122941; doi:10.1093/gbe/evu143)

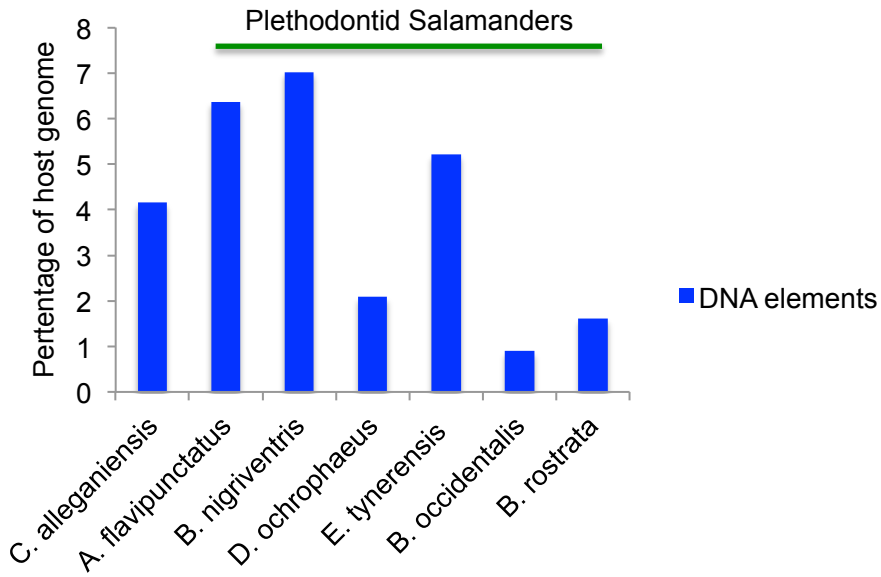

Supplement: Supplementary Data [file supp_evu143_AdditionalFile3.pdf]

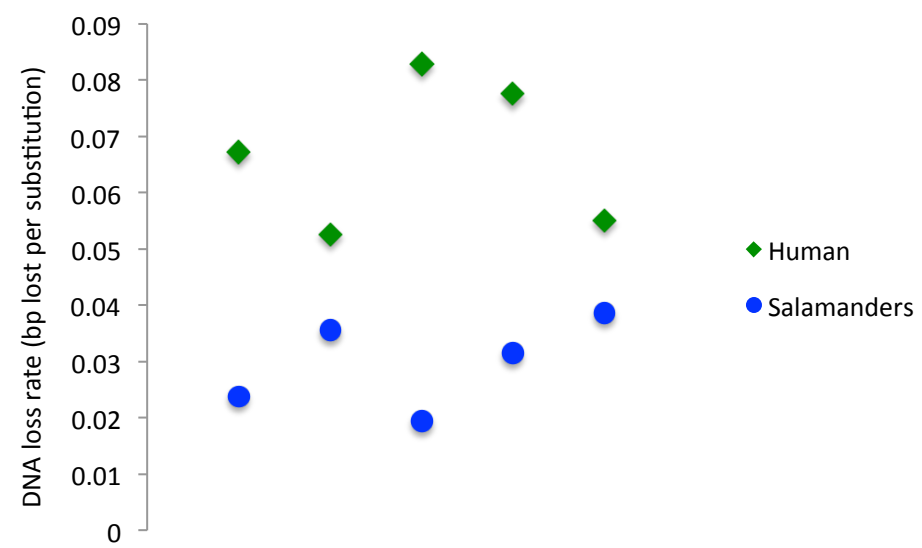

Supplement: Supplementary Data [file supp_evu143_AdditionalFile4.pdf]
